# The Continuous Motion Technique for a New Generation of Scanning Systems

**DOI:** 10.1038/s41598-017-07869-3

**Published:** 2017-08-04

**Authors:** Andrey Alexandrov, Annarita Buonaura, Lucia Consiglio, Nicola D’Ambrosio, Giovanni De Lellis, Antonia Di Crescenzo, Giuliana Galati, Valerio Gentile, Adele Lauria, Maria Cristina Montesi, Valeri Tioukov, Mikhailo Vladymyrov, Elena Voevodina

**Affiliations:** 1 INFN sezione di Napoli, I-80126 Napoli, Italy; 2 LPI - Lebedev Physical Institute of the Russian Academy of Sciences, RUS-119991 Moscow, Russia; 3 Universita’ degli Studi di Napoli Federico II, I-80126 Napoli, Italy; 4 INFN LNGS - Laboratori Nazionali del Gran Sasso, I-67100 Assergi, L’Aquila Italy; 5 GSSI - Gran Sasso Science Institute, I-67100 L’Aquila, Italy; 6 LHEP, University of Bern, Bern, Switzerland

## Abstract

In the present paper we report the development of the Continuous Motion scanning technique and its implementation for a new generation of scanning systems. The same hardware setup has demonstrated a significant boost in the scanning speed, reaching 190 cm^2^/h. The implementation of the Continuous Motion technique in the LASSO framework, as well as a number of new corrections introduced are described in details. The performance of the system, the results of an efficiency measurement and potential applications of the technique are discussed.

## Introduction

Nuclear photographic emulsions, also called Nuclear Emulsions, are the highest-precision tracking detector used in particle physics^1^. They are a rather cheap, compact and high-density device recording cumulatively all the charged particles passing through, being produced or stopped inside them. Unlike other detector types, nuclear emulsions must be developed before the tracks can be observed. After exposure and development single three-dimensional particle tracks can be observed and measured at a microscope, allowing reconstructing complex decay topologies especially important for short-lived decays.

Nuclear emulsion has typically the form of a glass plate or a thin plastic foil coated on one or both sides of a transparent support. It is made of silver halide micro-crystals immersed in a transparent organic gelatin compound. The energy loss of ionizing particles crossing the films induces along their path atomic-scale perturbations that, after a special chemical treatment called development, produce a sequence of silver grains in the emulsion visible through an optical microscope.

The history of the photographic method in particle physics began in 1896 with the discovery of radioactivity and since then it played an important role in particle and nuclear physics and astrophysics. Nuclear emulsions have set a milestone in the study of neutrino physics, in particular in the field of neutrino oscillations and for the first direct detection of the tau-neutrino. The improvements in the technology of emulsion readout has allowed to extend the application of nuclear emulsions to the dark matter search^2, 3^. The possibility to measure nuclear emulsions with faster scanning devices has provided a complementary and, when real-time monitoring is not required, even an alternative technique to electronic detectors in the field of muon radiography/tomography^4^ in particular for the study of the inner structure of volcanoes^5^ or the investigation of geological faults. Nuclear emulsions find interesting applications in hadron-therapy, where the use of carbon beams is limited by the poor knowledge of the secondary fragments emitted on the irradiated tissues. The added value of using nuclear emulsions for the study of carbon beams and their secondary particles has been demonstrated in several works^6–8^.

The first concept of automatic scanning system was proposed in 1974 at Nagoya University in Japan^9^, but at that time the digital technology was too primitive to implement it. It was not before 1990 when the first fully automated emulsion scanning system, named the Track Selector (TS)^10^, emerged in Japan. Upgraded versions of this system, called New-TS (NTS) and Ultra-TS (UTS) were used in the DONuT^11^ and CHORUS^12^ experiments. The latest version, called Super-UTS^13^ (S-UTS) was used in the OPERA experiment^14^. Since the early 90 s an independent automatic microscopy R&D program started in Italy, aimed at the development of systems for analysis of large emulsion surfaces for neutrino oscillation experiments (CHORUS and later OPERA) designed to observe muon-to-tau neutrino oscillations in appearance mode^15–19^. The efforts were finalized with the development of the SySal^20^ scanning system that later evolved into the European Scanning System^21, 22^ (ESS). Both the ESS and the S-UTS, used in the OPERA experiment, had high efficiency (about 90%) for MIP particles search and similar tracking resolution (1 micron in position and 3 mrad in angle in a 300 *μ*m thick film). The peak scanning speed achieved was of 20 cm^2^/h for ESS and 72 cm^2^/h for S-UTS with an angular acceptance up to 0.6 radian, satisfying the OPERA requirements. The data volume acquired by the automated microscopes for OPERA experiment is unprecedented in the world and equivalent to several thousands square meters of the emulsion surface (50 *μ*m thick) completely analyzed by systems developed in the experimental physics groups.

**Figure 1:**
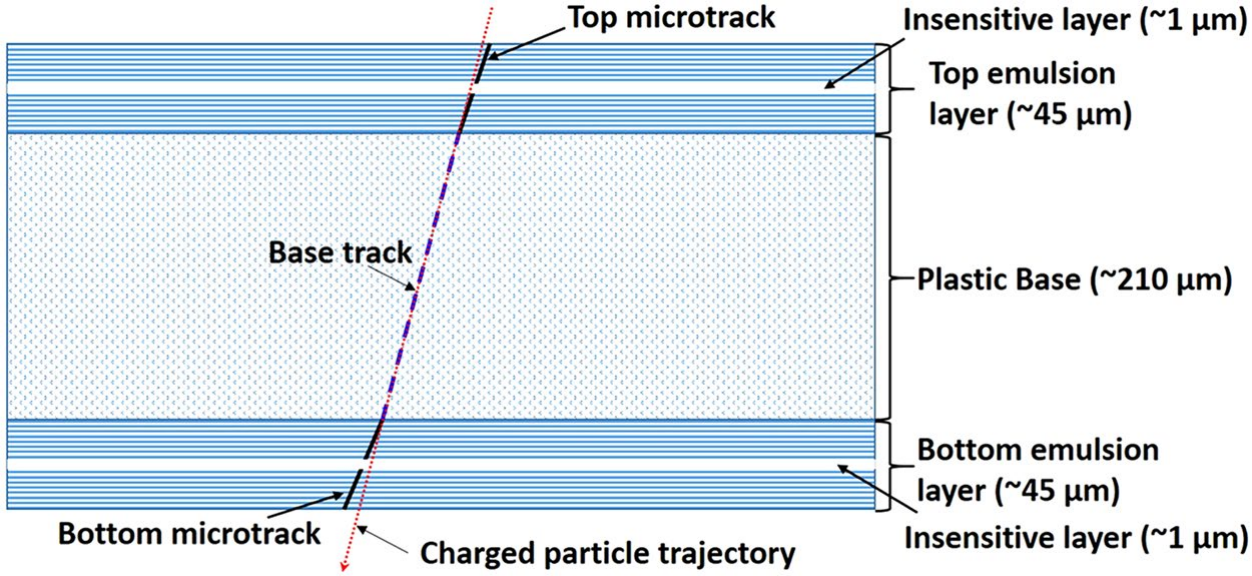
Passage of a charged particle through an OPERA-like emulsion film.

Schematic view of an OPERA-like emulsion film^23^ is shown in Fig. 1. It is composed of two emulsion layers, one top and one bottom, poured on either side of a plastic base. Due to the technological process of emulsion film production each emulsion layer contains in the middle a thin insensitive layer of pure gelatin. A passing through charged particle leaves traces in both sensitive emulsion layers visible as sequences of aligned silver grains and referred to as top and bottom microtracks. The measured direction of a microtrack may not coincide with the real charged particle trajectory due to the shrinkage effect: change of emulsion layer thickness during development. In OPERA-like emulsion films the shrinkage effect is minimized by filling them with glycerin after development, thus, almost precisely restoring the original thickness. After reconstructing a microtrack it is possible to trace it until the point where it crosses the plastic base surface. This point is the least affected by distortions and, therefore, lies closest to the particle’s trajectory. By interconnecting the least distorted points for top and bottom microtracks one obtains the best approximation of a passing-through particle trajectory, referred to as a base track.

Since 2011 the Naples OPERA group has carried out a dedicated R&D on automatic scanning^24^ to improve the performance of the ESS developing the Large Angle Scanning System for OPERA framework (LASSO)^25^ able to work up to 40 cm^2^/h on the current ESS hardware set-up and to measure tracks in emulsion in an extended angular range. In 2015 the ESS has been upgraded to the New Generation Scanning System (NGSS)^26^ that can operate with high efficiency at the record speed of 84 cm^2^/h. Different developments were carried out by other groups^27^. In this paper we report a further scanning speed improvement by the development of the Continuous Motion (CM) scanning technique and its implementation at the NGSS. With the development of the CM technique we have boosted the NGSS’s scanning speed to 190 cm^2^/h.

## Results

### Scanning techniques

The scanning method consists of taking a series of tomographic images while moving the focal plane of the objective inside the sensitive emulsion layer and in this way digitizing the full content of the sample. After the image processing and the three-dimensional reconstruction, one obtains shapes and positions of all silver grains. Images are taken with steps shorter than the objective’s depth of field: in this way the digitization and analysis of each cubic micron of sample is performed without gaps. The huge amount of information (up to several GB/s) coming from fast digital cameras requires high performance hardware, together with advanced image processing and computing algorithms. The image taking is synchronized with the motion of the microscope motorized stage and the objective lens (XYZ axes).

### The Stop&Go approach

**Figure 2:**
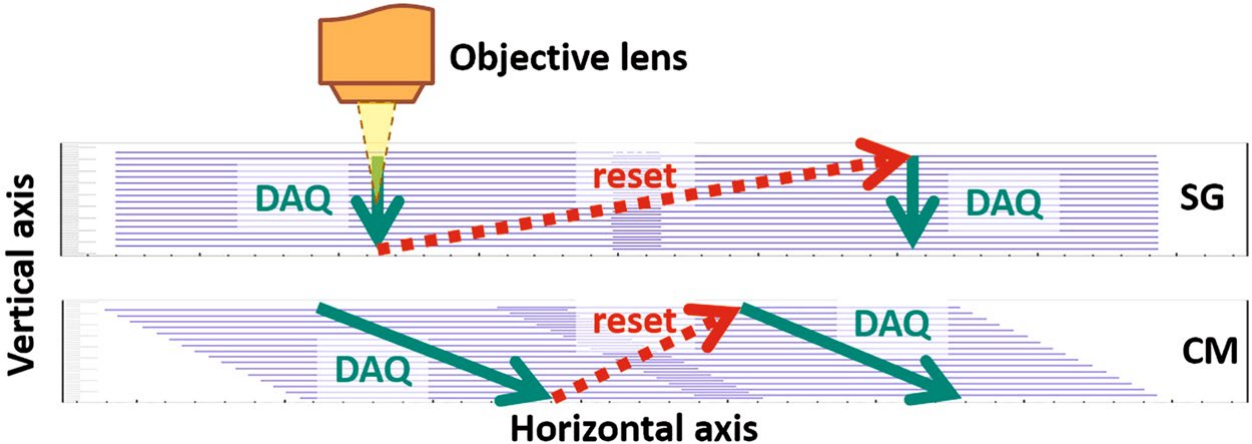
The schematic representation of the Stop&Go (SG) and the Continuous Motion (CM) scanning techniques.

The Stop&Go (SG) approach for the movement of the microscope stage and objective lens has been used for automatic scanning of emulsion films since the very beginning of the automated microscopes era. It has proven itself as a very reliable and relatively easy technique. In some sense it is the most straightforward way to implement the stage motion and it provides a set of images stacked vertically that is, in turn, the most convenient format for processing. The SG technique is described in details in ref. 26. The technique includes two steps called the data acquisition (or DAQ) motion and the reset motion. The DAQ motion involves only vertical movement of the objective lens and, therefore, depends only on the camera frame rate and the desired sampling step (the distance along the Z-axis between two consecutive frames). The reset motion is intended to move the objective and the stage to the next field of view. Therefore, it involves movement along both vertical and horizontal axes, and the required time is then defined by the longest movement. Since in most of the applications the emulsion thickness (tens of *μ*m) is several times smaller than the field of view dimensions (hundreds of *μ*m), the longest movement is the horizontal one. As it is shown in the Fig. 2 (top scheme), during the DAQ phase of the SG (green solid arrow) the objective lens moves with a constant speed along the vertical axis only, producing a vertical image pile (blue thin horizontal lines), and then performs a long reset motion (red dotted arrow) to the next field of view. With wide fields of view and thin emulsions the reset motion takes even longer than the DAQ motion. In a certain sense the reset motion can be interpreted as a “dead time” of the microscope since no measurements are carried out during it. With the technological progress it becomes possible to construct automatic microscopes with even wider fields of view and shorter DAQ motion time by choosing lower magnification objective lens along with cameras having more (mega-)pixel sensors and higher frame rates. The use of the SG technique at such microscopes would only increase the “dead time” fraction of new systems.

### The Continuous Motion approach

In order to fully exploit the hardware components of the microscope we have developed a novel scanning approach called the Continuous Motion (CM) reported in this paper. In this approach, shown schematically in the Fig. 2 (bottom scheme), the vertical axis performs a periodic movement while the horizontal one moves at a constant speed, such that during one period of the objective lens oscillation the stage displacement amounts to exactly one field of view. Then, the size of the overlap of two consecutive fields of view can be increased by decreasing the horizontal speed.

If one considers an emulsion layer with the thickness *d* and a scanning system equipped with a stage capable of moving the objective lens with the acceleration *a*_*z*_ and a camera capturing *f* frames per second, then the time *T*_*CM*_ required to scan a single view with Z-sampling *s* can be expressed with the formula:

1 $${T}_{CM}=2\frac{{v}_{z}}{{a}_{z}}+\frac{d}{{v}_{z}}+2\sqrt{\frac{d+{v}_{z}^{2}/{a}_{z}}{{a}_{z}}},$$

where the first two terms correspond to the DAQ phase and the last one to the reset motion phase. The first term describes the time needed to accelerate to the speed *v*_*z*_ = *sf* and the factor 2 accounts for the equal time needed for deceleration. The second term represents a movement with a constant speed *v*_*z*_ through the emulsion thickness *d* during which frames are actually grabbed. The last term corresponds to the acceleration time over the distance $(d+{v}_{z}^{2}/{a}_{z})/2$ and the equal deceleration time is accounted for by the factor 2. The formula can be simplified to take the form:

2 $${T}_{CM}={(\sqrt{\frac{d+{v}_{z}^{2}/{a}_{z}}{{v}_{z}}}+\sqrt{\frac{{v}_{z}}{{a}_{z}}})}^{2}={(\sqrt{\frac{h}{sf}}+\sqrt{\frac{sf}{{a}_{z}}})}^{2},$$

where $h=d+{v}_{z}^{2}/{a}_{z}$ is the overall scanning amplitude including the emulsion thickness *d* as well as the space above and below it needed to accelerate and decelerate the objective lens.

Unlike the SG, in the CM a tilted set of images is produced during the DAQ step, with every image being displaced horizontally by a certain distance with respect to its neighbors. A tilted image set requires more sophisticated processing, compared to the SG, since most of the tracks will cross view boundaries as well as grain images (referred to as clusters) belonging to the same track will appear at different positions within different images. Thus, the processing must take into account effects of optical distortions, vibrations and views misalignment.

### Implementation of the Continuous Motion technique

The idea of the CM is to let the horizontal stage move at a constant speed along one of the axes while the vertical stage performs the DAQ and reset motions. Thus, the most straightforward implementation would be to command the horizontal stage to start moving and then control only the vertical stage by sending fast commands to move up and down at the desired speed and acceleration.

Unlike the SG, the CM is very sensitive to any kinds of delay in the commands flow and processing. Indeed, with the typical working cycle of 80 ms the horizontal speed will be around 10 mm/s. Thus, a delay of 1 ms would lead to about 10 *μ*m of extra stage displacement to be taken into account in the overlap. The Windows XP used to control the stage PC is essentially not a real-time operating system. It has a task scheduler that is not under user control and a typical time slice of the order of 20 ms. So a delay of several tens of ms can easily occur resulting in up to several hundreds of *μ*m of extra stage displacement. This displacement must be taken into account in overlaps otherwise gaps between adjacent views can appear. Overlaps of several hundreds of *μ*m would vanish all the advantages of the CM, canceling out any significant gain in the scanning speed.

To guarantee the delay-free commands flow we use the programmable FPGA device present on the NI-Motion PCI-7344 stage controller board. Commands and their parameters can be stored in an onboard buffer without interrupting the ongoing stage motion. The next command is executed immediately after the previous one is accomplished, with no delay. The FPGA API provides functionality for looping and branching, as well as functions to query the current motion status. It allows to write simple standalone programs that can be executed onboard without interfering with the main CPU.

**Figure 3:**
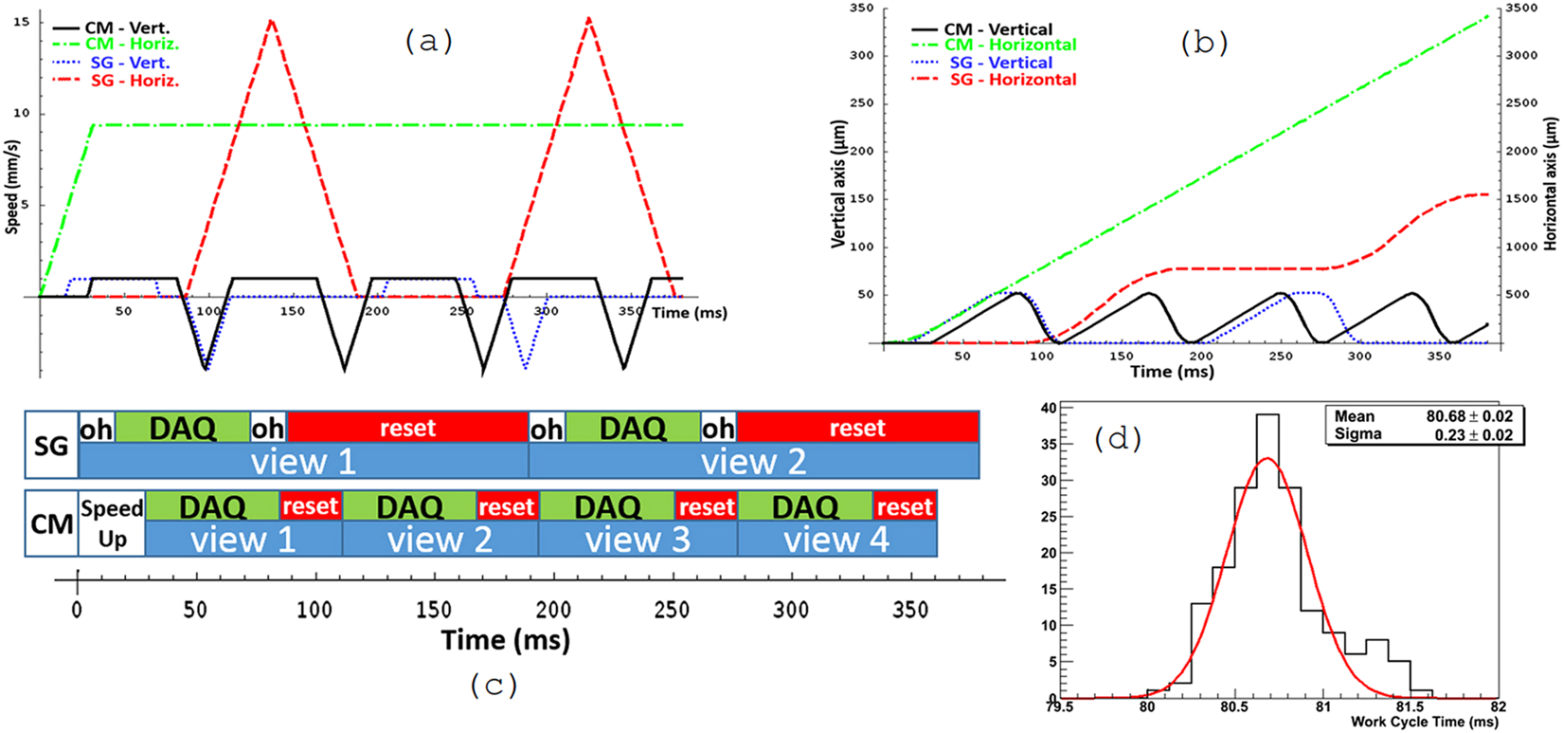
Speed (**a**) and displacement (**b**) profiles and scanning phase chart (**c**) for the SG and the CM techniques. The DAQ phase takes the same time in both techniques while the reset time is drastically reduced in the CM. The plot (**d**) shows the working cycle time distribution for the FPGA implementation of the CM.

Following this approach, the FPGA is programmed to execute the DAQ motion followed by the reset motion in an infinite loop, making the objective oscillate up and down with a stability of the period better than 1 ms (see Fig. 3d). This introduces the uncertainty of less than 10 *μ*m to be taken into account in overlaps. The possibility to preload the next movement parameters before the current one is completed makes moves follow one after each other with negligible delays, thus bringing the overhead time to practically zero. The comparison of the speed and displacement profiles of the SG and CM techniques is shown in Fig. 3a and b. The DAQ and reset phases duration is shown in the Fig. 3c. The corresponding scanning parameters are reported in Table 1.

**Table 1:** Comparison of LASSO scanning parameters and performances between the SG and the CM techniques.

Scanning Technique	**SG**	**CM**
Camera frame rate (fps)	563	
Field of view (*μ* × *μ*m)	805 × 595	
Pixel to micron ratio (*μ*m/pixel)	0.34	
Sampling step (*μ*m)	1.75	
Frames per view	28	
Scanning depth (*μ*m)	49	
Views overlap (*μ*m)	30	
DAQ time (ms)	57	
Reset time (ms)	102	24
Overhead time (ms)	30	0
Working cycle (ms)	189	81
Scanning speed (cm^2^/hour)	84	190

### Continuous Motion workflow

The CM technique was implemented within the LASSO framework^25^. The latter has a modular structure with a number of modules each having a definite role: the Stage Module controls stage movements and position monitoring; the Camera Module performs image acquisition with frame-grabber and camera; the Image Processing Module processes images in real-time using available GPU boards; the Tracker Module performs real-time reconstruction of microtracks; the Guide Module ensures reliable co-operation of all modules and governs the scanning process.

**Figure 4:**
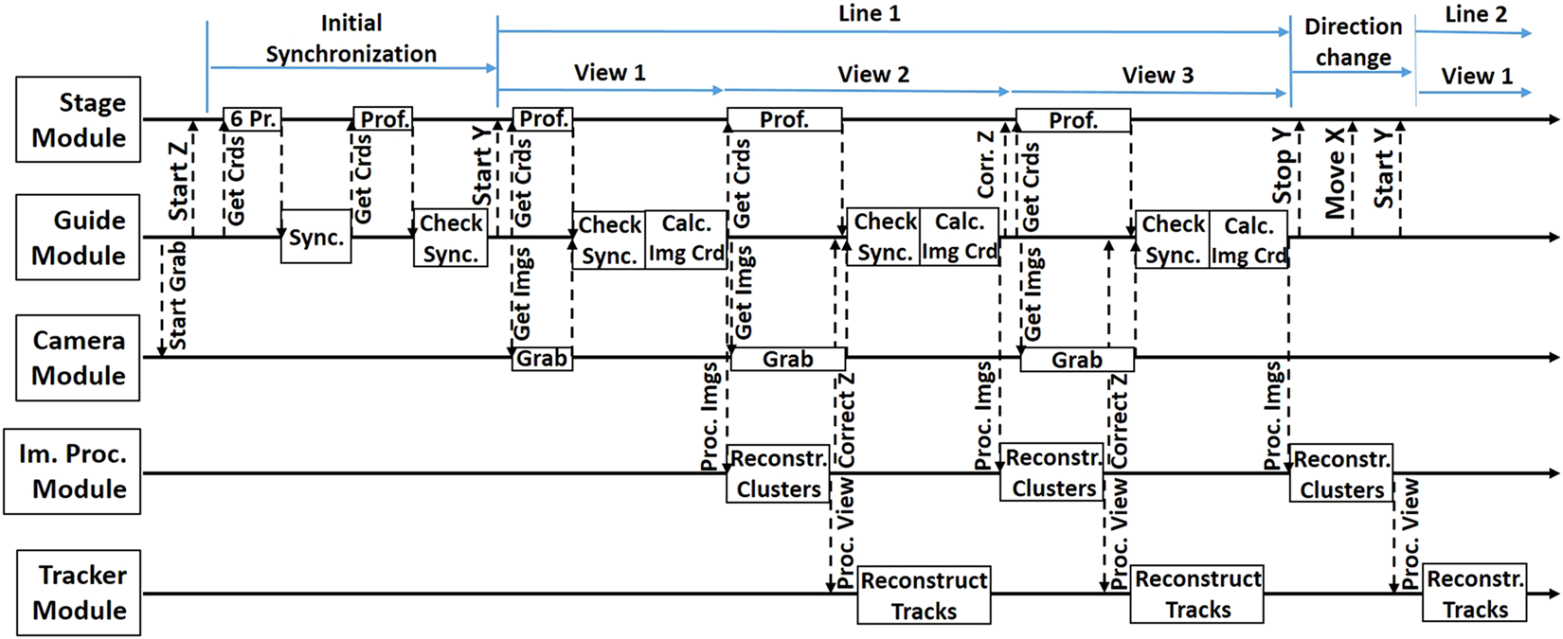
Workflow diagram for the CM technique. Thick solid horizontal arrows indicate the execution flow. Thin dashed vertical arrows indicate commands flow directed from client module to server module that executes it. Rectangular boxes represent operations requested.

During the operation, different modules exchange data by issuing commands and waiting for responses. The workflow diagram is shown in Fig. 4. The scanning process is governed by the Guide module that starts with issuing a command to the Camera module to start image acquisition. The Camera module grabs images and stores them into a circular buffer overwriting the oldest images. The buffer length is adjustable and typically it contains 1000 images, large enough to hold about 1.75 seconds of data.

After the image acquisition is started the Guide module commands the Stage module to start oscillating along the vertical axis and requests the movement profile for 6 periods (the “6 Pr.” operation in Fig. 4). After the Stage module has provided the displacement profile (black solid line in Fig. 3b) the Guide module synchronizes with vertical oscillations by isolating individual DAQ and reset phases. From now on it is capable to predict when DAQ phases will take place even for several periods in advance. In order to check that the synchronization was calculated correctly, the Guide module calculates the nearest DAQ window and requests the Stage module to provide displacement profile for that period (the “Prof.” operation). This check (the “Check Sync.” operation) is carried out every time when position data arrives to ensure the validity of the acquired data.

After the completion of the synchronization routine the movement along the horizontal axis is started. The Guide module calculates when the nearest DAQ phase occurs and issues requests for motion profile (“Prof.”) and images (“Grab”) for the corresponding time window. As soon as the DAQ phase is over, it receives the requested images and coordinates, checks synchronization and calculates image coordinates (“Calc Img Crd”). Then it sends images to the Image Processing module that performs cluster reconstruction and location of emulsion surfaces. The latter is required to control that the data is taken inside the emulsion sensitive layer and, if due to local curvature or thermal expansion the vertical position of the emulsion changes, the Image Processing module issues a feedback to the Guide module that oscillation limits for the vertical axis should be corrected. Reconstructed clusters are sent to the Tracker module that performs further processing: reconstruction of grains, alignments and reconstruction of microtracks.

### View reshaping by merging of the adjacent views

**Figure 5:**
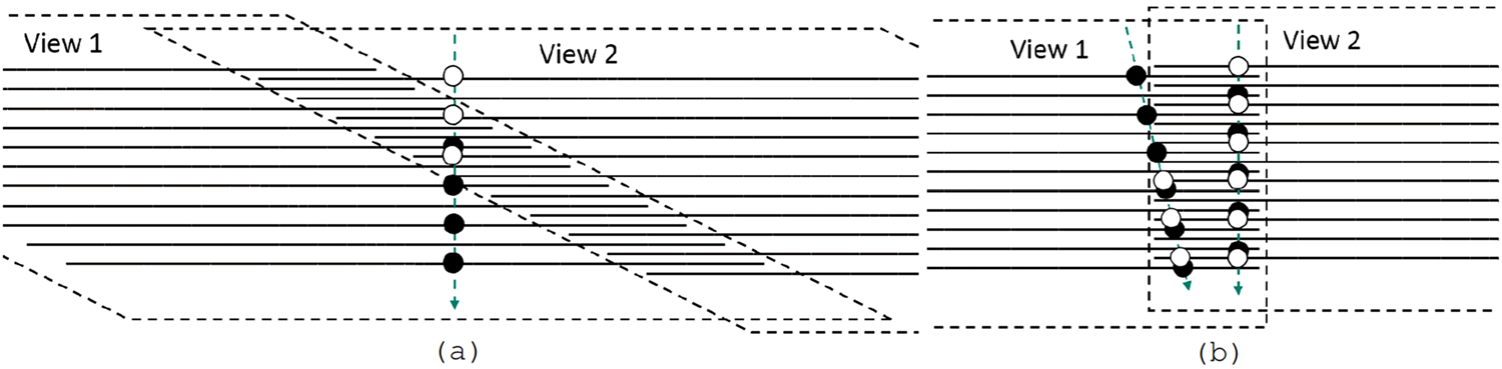
Tracks crossing adjacent views, made of grains in tilted (**a**) and vertical (**b**) overlapping piles of images. Dashed arrows represent particle tracks. Solid horizontal lines represent images. Circles represent images of grains detected along the track. Black (white) circles are clusters detected in the left (right) view.

As it was mentioned before, in the CM a tilted pile of images is produced leading to a reconstruction volume of non-rectangular shape. With such a shape a significant fraction of vertical tracks will only be partially contained in the reconstructed volume. For example, shown in Fig. 5a, the three bottommost (black) clusters are detected only in the left view, while the two uppermost (white) clusters are detected only in the right view. Thus, the track is only partially contained in either view, becoming split into two rather short segments. Partially contained tracks have a higher probability to be lost during the reconstruction process. This problem can be minimized if the reconstruction volume has a rectangular shape with vertical sides. In this case, shown in Fig. 5b, if a quasi-vertical track exits through a side then due to overlapping of volumes it will be reconstructed in the adjacent volume. Similarly, an inclined track crossings the boundary will be reconstructed in at least one volume since its travel path inside will be long enough.

**Figure 6:**
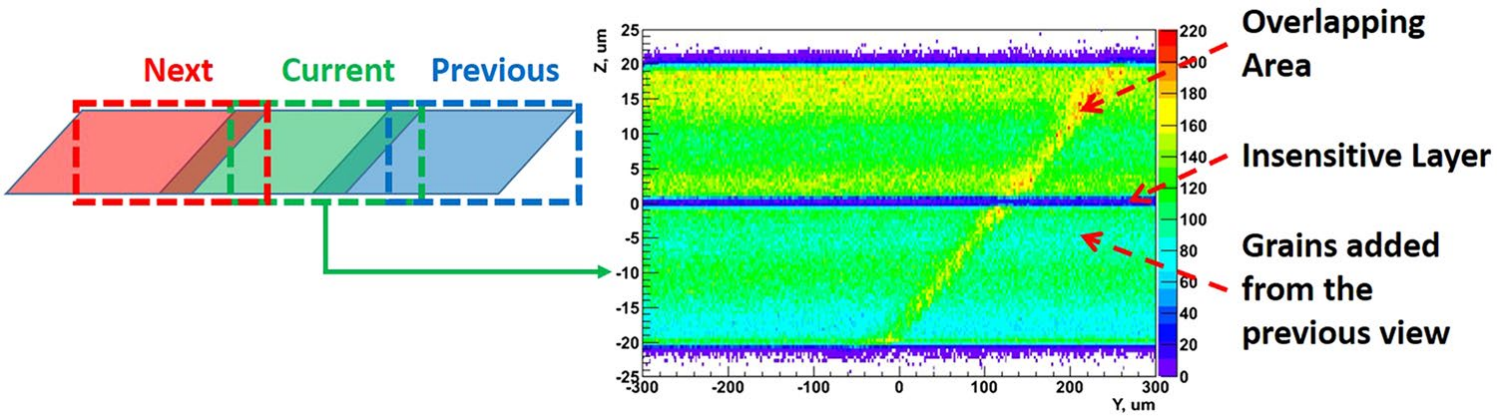
Illustration of the view reshaping procedure.

In order to reshape the volume we first reconstruct grains inside it and then align it with the previously processed adjacent volume by matching grains in the overlapping area. Then all the grains belonging to the adjacent volume and falling inside the reshaped volume are added to it. The precision of the grain reconstruction may decrease if a grain is within 10 *μ*m from the edge of the view since a part of it can lie outside and the grain is only partially contained. On the other hand, its duplicate is fully contained in the area of overlap with the adjacent view. Therefore, marginal grains are discarded and do not take part in the matching procedure. The matching grain pairs, detected in both volumes, are merged into single grains. After that a newly formed rectangular shape volume undergoes the usual microtrack reconstruction procedure, the same one as for the SG. The procedure of view reshaping is illustrated in Fig. 6.

### Optical distortion correction

**Figure 7:**
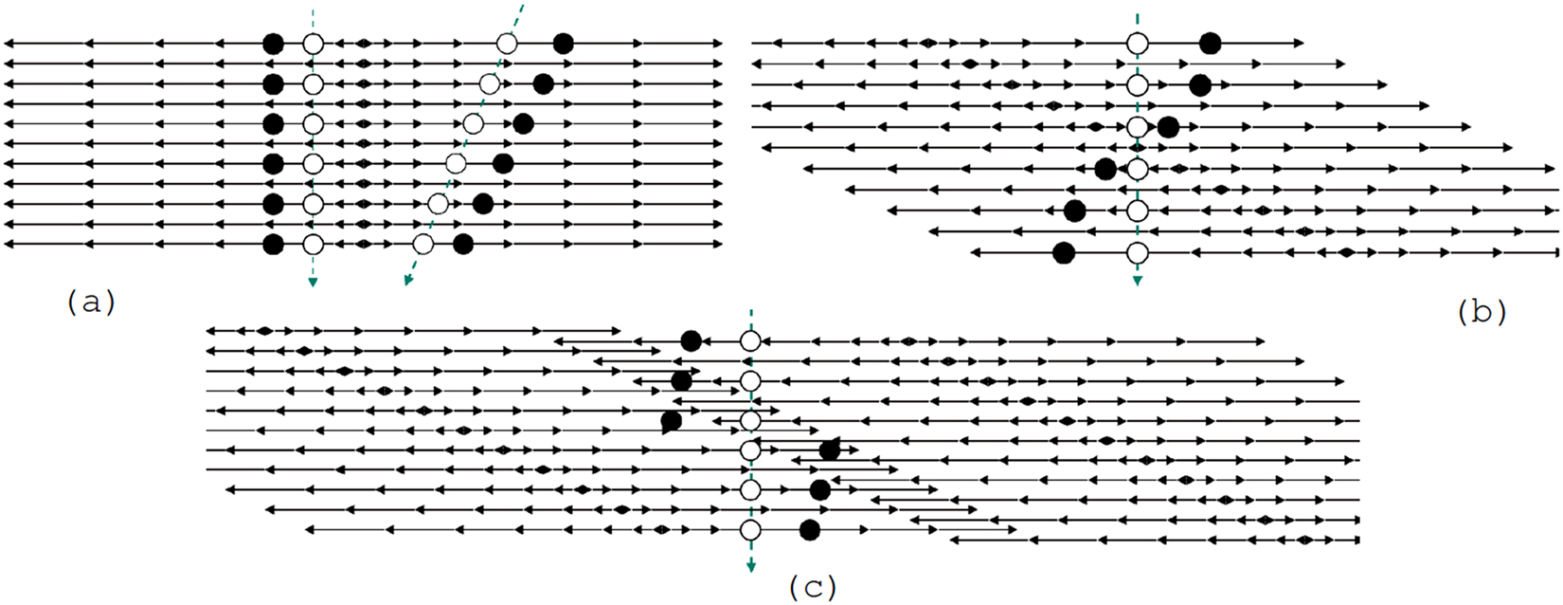
Distortion of a vertical and an inclined track passing through emulsion in the SG (**a**). Distortion (**b**) and breakage (**c**) of a vertical track passing through emulsion in the CM. In all figures horizontal lines represent grabbed images. Solid horizontal arrows represent distortion strength and direction. Dashed arrows represent particle tracks. Empty circles represent true positions of grains left by passing through particles. Black circles represent the observed grains positions as they are seen due to optical distortions.

Optical distortion, inevitably present in any optical system, can spoil microtrack reconstruction in emulsion. This effect leads to discrepancy between the true position of a grain and its visible position inside the field of view. It is position dependent and reaches the maximal value (up to 1–2 *μ*m) near the field of view edges, affecting all three coordinates. Since in the SG all the frames are piled up vertically, grains of a moderately inclined track, which is usually the case, appear more or less in the same part of the field of view, thus experiencing more or less equal distortion (see Fig. 7a). Therefore, all grains of the track are equally shifted giving rise to a coherent displacement of the microtrack from its true position, but it does not affect the shape of the microtrack. So in the SG the optical distortion does not create problems other than some position accuracy degradation. On the contrary, in the CM with its tilted image pile, grains of the same track, even a vertical one, appear in different parts of the field of view undergoing distortions of different magnitude and direction (Fig. 7b). Therefore, optical distortion, if not corrected, changes the shape of a straight track curving it and even breaking it if it passes through the tilted side of the volume (Fig. 7c).

In order to get rid of optical distortion effects we have introduced two correction matrices: one performs correction in the horizontal (XY) plane while the other one along the vertical (Z) axis. The use of two matrices instead of a single three-dimensional one was dictated by reasons of implementation. The distortion correction procedure is described in details in ref. 25.

### Camera time offset correction

The timestamp *τ* of an image is associated to the coordinate of the image centre *ξ* through a procedure described in the Methods Section. Nevertheless, the timestamp is not perfectly synchronized to the moment when the image is centered around *ξ*. Note that the difference between consecutive timestamps is very precise. As long as we move in one direction, the displacement of view centers with respect to their true position is always the same. On the contrary, as soon as we change direction of movement, this displacement changes its sign. This causes the hysteresis effect. In the SG this effect was avoided by performing the data acquisition always in one and the same movement direction of the vertical axis. The same technique is used in the CM but for vertical axis only. Making it for horizontal axis would lead to a significant drop in the scanning speed. Therefore, the only way to get rid of hysteresis is to introduce the timestamp offset correction, a value that would be added to every image timestamp thus fixing it and giving it a certain sense as the moment when the central pixel is acquired.

### Fine vibration correction by view-to-view alignment

The existing vibration correction procedure performs alignment of the adjacent frames taking advantage of grain shadows. Thus, it corrects vibrations inside the view only, leaving uncorrected view’s coordinates with respect to neighboring views. In other words the correction is local. To make it global we developed a procedure that aligns each view with its available neighbors.

The alignment is performed at the grain level, i.e. it uses all the three coordinates of a reconstructed grains. The procedure searches for matching grain patterns in the overlapping volume of neighboring views. To improve alignment accuracy and stability some redundancy can be added by aligning with views from the previously scanned line. Therefore, the entire line of views is stored on purpose in the computer memory. In order to save memory space, the program keeps only peripheral grains close to the view boundaries. The alignment is done online and found offsets are saved for later use in a dedicated offline procedure that merges all the views into a single volume before reconstructing tracks.

View alignment also improves the timestamp correction precision: after traveling for a long distance even a small discrepancy can accumulate view by view and give a noticeable offset degrading the position resolution. Due to this offset microtracks in the overlapping area corresponding to the same track appear too far apart to be recognized as a matching pair, leading to the appearance of fake tracks. The view alignment between the current view and its closest neighbors helps reduce this effect.

### Thermal expansion correction by alignment with reference views

In case of double sided emulsion films thermal effects become important. During scanning of one emulsion layer, the stage heats up and expands, thus, displacing emulsion layer vertical positions. If measured directly from the data, the distance between layers is not equal to the plastic base thickness as it should be. After one hour of scanning the discrepancy can be as large as 50 *μ*m and, if not corrected, leads to incorrect measurement of track slopes and, hence, to the degradation of the angular resolution and efficiency. Irregularities in emulsion film flatness and base thickness are usually much smaller than the thermal expansion and do not create problems neither during scanning nor in analysis. The solution used in the SG is to divide emulsion area into fragments small enough that during scanning of one emulsion layer inside the fragment, the second one would not have any noticeable displacement. This solution does not affect the scanning speed in the SG, but in the CM it would significantly decrease it due to the necessity to switch more often between emulsion layers as well as change the scanning direction.

In order to correct the effect of thermal expansion without decreasing the scanning speed, we have introduced the concept of reference views: view pairs (one top and one bottom) separated horizontally by few centimeters. The acquisition of reference views is performed at the start of data taking and takes no longer than a minute and, therefore, emulsion layer’s displacement during it is negligible. As soon as reference views are taken the emulsion film is scanned as a single fragment. Later all the views are aligned between each other as well as with reference views thus providing information for the calculation of a global transformation. The transformation, being applied to the view coordinates, moves the view to the position it was during the reference view scanning operation, thus restoring the original form of the emulsion film correcting the effect of thermal expansion.

## Discussion

The presented work reports the development of the CM scanning technique. With this technique it has become possible to boost the NGSS microscope’s effective scanning speed from 84 cm^2^/h to 190 cm^2^/h, thus setting a new record. The increase in the scanning speed is due to the reduction of the reset motion time needed to move the objective and the stage to the next field of view. The high performance level was achieved thanks to the development and application of a number of correction procedures: optical distortion correction, timestamp offset correction, vibration correction by view alignment and thermal expansion correction by alignment with reference views. It has become possible to use the existing microtracking procedure without changes after implementing the view reshaping by merging grains from the previous view.

**Figure 8:**
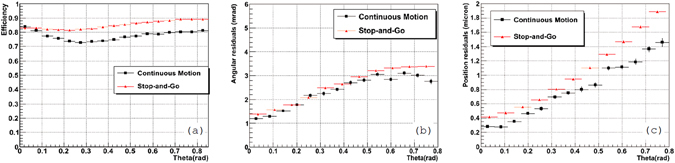
Base track reconstruction efficiency (**a**), angular residuals (**b**) and position residuals (**c**) versus track angle measured with a NGSS microscope.

The efficiency test was performed using a stack of OPERA-like emulsion films. Due to increased vibration level, in-motion acquisition and view reshaping, reconstructed grain positions in the CM case are less accurate. If one tries to compensate that by loosening parameters in the microtrack reconstruction procedure, the chance coincidence level will significantly increase giving rise to the purity degradation. For this reason microtracks reconstruction was done with the same parameters as in the SG. Naturally, that led to some decrease in the reconstruction efficiency that had propagated through all other reconstruction stages and is visible in Fig. 8a. On the other hand, the application of the view-to-view and reference view alignments at later stages have recovered angular and spatial accuracies of the base track reconstruction as shown in Fig. 8b and c, resulting in higher reconstruction quality. Angular residuals are calculated as an average angular difference between two consecutive base tracks associated with the same reconstructed track. Position residuals are calculated as an average difference between the position of a base track in one film and the projection of a consecutive base track to the same film. It is worth noting that the reconstruction efficiency of a single base track is not critical due to the redundancy provided by having 10 or more emulsion films along the particle trajectory, therefore, the measured performance is found to be fully satisfactory and comparable to that in the SG.

The NGSS microscope described in this paper is currently in use for the final analysis of the OPERA films. The development and implementation of the proposed CM technique allows to reach a scanning speed one order of magnitude higher than in OPERA, thus enabling the achievement of much more challenging goals. Unlike the SG, the CM enables the use of latest technological advances for further scanning speed improvement, e.g. the use of a piezo-driven vertical stage or multiple cameras.

The CM technique can be fruitfully applied to any application of the scanning of nuclear emulsion films in high energy, astroparticle and nuclear physics. In particular we see the following applications:

- The FOOT^28^ (FragmentatiOn Of Target) experiment is designed to study the interactions of carbon ion and proton beams in the patient tissues in order to optimize the hadron-therapy treatment planning systems. Its detector is based on the use of nuclear emulsion to detect light fragments emitted at large angles;
- The NEWSdm^2^ (Nuclear Emulsions for WIMP Search with directional measurement) experiment designed to search for dark matter candidates in the underground Gran Sasso Laboratory by using the innovative approach of detecting the nuclear recoil direction with an emulsion target;
- The neutrino detector of the SHiP^29^ experiment that will use large amount of emulsion films as a tracking detector to study tau neutrino physics and search for light dark matter produced by 400 GeV proton interactions;
- The muon radiography/tomography to study the inner structure of volcanoes and geological faults, where emulsion films, unlike electronic detector, can be easily installed and large surfaces are required;
- The CM technique can also be used with samples different from emulsion films, e.g. biological samples, where large volumes have to be analyzed with optical microscopes in the shortest possible time.

## Methods

### Microscope setup

The NGSS microscope setup is equivalent to that described in ref. 26.

### Emulsion sample

The CM performance has been studied on a stack of OPERA-like emulsion films exposed at CERN to a 6 GeV/c *π* ^*−*^ beam. The films were exposed several times with different incident angles. The track density per each angular peak was determined to be around 2000 particles/cm^2^.

### Scanning configuration

We have scanned an emulsion surface of 30 cm^2^ in 8 consecutive plates. The final configuration of the scanning parameters to gain the maximum efficiency has been determined choosing 28 layers of tomographic images taken with a Z-sampling of 1.75 *μ*m, a 5 × 5 high-pass convolution filter and a pixel-dependent binarization threshold applied. The effective scanning speed was 190 cm^2^/hour taking into account overlap of adjacent views (about 30 *μ*m) and the time required to scan 9 reference views. For other scanning parameters please refer to Table 1.

### Track reconstruction

The track recognition procedure is a quite complex process executed by the LASSO software tracking module: all the steps of the algorithm for the microtrack reconstruction are described in ref. 25. Then a dedicated offline software FEDRA^30^ performs the track reconstruction in the full volume data: after the connection of microtracks across the plastic base (to form base tracks), which strongly reduces the number of fake tracks in a single film, a plate to plate alignment is done to connect base tracks in consecutive sheets and recognize the volume tracks. In such a procedure, most of the instrumental background tracks are discarded. The base track efficiency has been finally evaluated as the number of segments belonging to passing-through tracks divided by the number of crossed plates except the first and the last ones where each track starts and ends respectively.

### Horizontal distortion correction

To obtain the horizontal (XY) matrix we perform scanning of approximately 80 × 60 views at one and the same depth inside emulsion with horizontal step of 10 *μ*m. In this way we obtain a dataset where an image of a grain (a cluster) appears in different parts of the field of view thus being distorted in different ways. Then we identify that grain in all images by performing a pattern matching between clusters. The identification procedure is repeated for every grain. Then it is possible to compare the displacement of the grain from the position predicted by the stage encoder. This difference constitutes the desired local correction convoluted with stage vibration. Therefore, the scanning is performed with lowest possible speed and acceleration. The field of view is subdivided into cells, with the number of cells being equal to the camera resolution. The effect of vibrations is further reduced by averaging individual corrections for grains that fall inside the same cell. These cells form the horizontal correction matrix that later is applied to every found cluster displacing it in the XY plane according to the values computed for the corresponding cell.

### Vertical distortion correction

To calculate the vertical (Z) matrix we profit of the OPERA emulsion film structure: due to technical reasons it is produced with a 1 *μ*m insensitive layer of pure gelatin between two sensitive layers (see Figs 1 and 6). Typically, an area of several cm^2^ is scanned and grains are reconstructed. In order to determine the distorted shape of the insensitive layer we subdivide the field of view into vertical columns, each being 10 × 10 *π*m^2^ in XY and spanning the whole Z. These columns are filled with the grain coordinates relative to their original views. Then the Z coordinate of the insensitive layer is found for each column by looking for the minimum grain density therein. The set of found values provides the vertical distortion correction matrix.

### Camera time offset correction

The calculation of the time offset is performed during the microscope setup and tuning. During the normal scanning operation only the offset value is used. To find the offset value we scan the same area inside emulsion twice in opposite directions. Then alignment is performed by matching grains reconstructed in both datasets. The time offset can be calculated with the formula *dt* = *dt*/(2*v*_*y*_), where *dy* is the offset in position of matched grains and *v*_*y*_ is the movement speed.

### Image coordinates calculation

The coordinates calculation is based on the timestamp information provided by the stage and camera modules. Sets of coordinates-timestamp pairs {*x*_*i*_, *t*_*i*_} for each axis constitute stage displacement profiles shown in Fig. 3b. To calculate image coordinate *ξ*, one searches for two points (*x*_*i*_, *t*_*t*_) and (*x*_*i*+1_, *t*_*i*+1_) with *t*_*i*_ < *τ* < *t*_*i*+1_, whekre *τ* is the image timestamp. Then one can use a simple interpolation formula *ξ* = *x*_*i*_ +(*τ* − *t*_*i*_)(*x*_*i*+1_ − *x*_*i*_)/(*t*_*i*+1_ − *t*_*i*_).

### Data availability statement

The datasets generated and analysed during the current study are available from the corresponding author on reasonable request.
